# Synergistic Spatial Confining Effect and O Vacancy in WO_3_ Hollow Sphere for Enhanced N_2_ Reduction

**DOI:** 10.3390/molecules28248013

**Published:** 2023-12-08

**Authors:** Yuzhou Xia, Xinghe Xia, Shuying Zhu, Ruowen Liang, Guiyang Yan, Feng Chen, Xuxu Wang

**Affiliations:** 1 College of Chemistry, Fuzhou University, Fuzhou 350116, China; yzxia@ndnu.edu.cn (Y.X.); 211327039@fzu.edu.cn (X.X.); 2 Fujian Province University Key Laboratory of Green Energy and Environment Catalysis, Ningde Normal University, Ningde 352100, China; rwliang@ndnu.edu.cn (R.L.); ygyfjnu@163.com (G.Y.); 3 State Key Laboratory of Photocatalysis on Energy and Environment, Research Institute of Photocatalysis, College of Chemistry, Fuzhou University, Fuzhou 350116, China; xwang@fzu.edu.cn

**Keywords:** WO_3_, hollow sphere, O defect, N_2_ reduction, photocatalysis

## Abstract

Visible-light-driven N_2_ reduction into NH_3_ in pure H_2_O provides an energy-saving alternative to the Haber–Bosch process for ammonia synthesizing. However, the thermodynamic stability of N≡N and low water solubility of N_2_ remain the key bottlenecks. Here, we propose a solution by developing a WO_3−x_ hollow sphere with oxygen vacancies. Experimental analysis reveals that the hollow sphere structure greatly promotes the enrichment of N_2_ molecules in the inner cavity and facilitates the chemisorption of N_2_ onto WO_3−x_-HS. The outer layer’s thin shell facilitates the photogenerated charge transfer and the full exposure of O vacancies as active sites. O vacancies exposed on the surface accelerate the activation of N≡N triple bonds. As such, the optimized catalyst shows a NH_3_ generation rate of 140.08 μmol g^−1^ h^−1^, which is 7.94 times higher than the counterpart WO_3_-bulk.

## 1. Introduction

Ammonia synthesis becomes one of the most important industrial reactions due to the widespread use of NH_3_ as fertilizers, pharmaceuticals, chemical feedstocks, and a promising clean energy carrier [1,2,3,4]. Up until now, NH_3_ production has greatly relied on the Haber–Bosch nitrogen fixation process, which was awarded as one of the most significant inventions of the 20th century. However, the reductant H_2_ used in this process is mainly derived from the steam reforming of biomass, and the reaction occurs under stringent reaction conditions (350–550 °C, 200–350 atm), which consumes up to ~2% of the total energy each year [5,6,7,8]. The excessive consumption of fossil energy and massive carbon dioxide emissions make this process not conducive to the sustainable development of mankind. Therefore, environmentally friendly NH_3_ synthesis via a renewable route is highly desirable [9,10].

Photocatalytic N_2_ reduction with H_2_O assembles the advantages of low cost, safety, and environmental friendliness to produce ammonia [11,12]. The mechanism of photocatalytic N_2_ reduction is as follows: N_2_ is first absorbed onto the surface of the photocatalyst. Due to the strong interaction between N_2_ and active sites, the sturdy triple bond is weakened, and the N_2_ molecule is activated. Under irradiation, the photogenerated electrons on the conduction band of semiconductors transfer to the activated N_2_ molecules, reducing N_2_ into NH_3_ with the participation of water. The evolved NH_3_ is desorbed from the catalyst surface. Since the pioneering work reported by Schrauzer on using Fe-doped TiO_2_, extensive efforts have been made to broaden the family of photocatalysts for N_2_ reduction [13]. So far, diverse catalysts have been developed for photocatalytic N_2_ reduction, including BiOBr, InVO_4_, Bi_2_WO_6_, Bi_2_Sn_2_O_7,_ and so on [14,15,16,17,18]. Despite much progress, the overall efficiency is still much less than satisfactory due to the bottlenecks of the extremely low solubility of N_2_ in H_2_O and the large dissociation energy of N≡N (941 kJ mol^−1^) [19,20]. To tackle these, morphology and structure optimization to regulate the physical and chemical properties of photocatalysts may be effective strategies [21,22,23,24,25].

Hollow materials illustrate distinctive physicochemical properties due to the unique structure of the confined thin shell layer and internal cavity [26,27,28], presenting as ideal candidates for photocatalytic N_2_ reduction. The spatial confining effect of the internal cavity is beneficial for restricting N_2_, enriching N_2_ molecules on the surface of the photocatalyst, thereby accelerating reaction kinetics. Moreover, the thin shell ensures the sufficient exposure of active sites. On the other hand, surface defects on photocatalysts with highly localized electronic structural changes are efficient active sites for N_2_ adsorption and activation [29,30,31,32]. Especially for the O defect, the electron-rich environment is beneficial for the cleavage of the N≡N triple bond via the π-back-donation into N_2_ antibonding orbitals, achieving a smoother hydrogenation process [33]. For instance, Mi and coworkers have reported the preparation of TiO_2_ with O defects via a solid phase reduction with NaBH_4_. The normalized N_2_ photofixation rate is 324.86 μmol h^−1^ g^−1^, which is 3.85 times that of the original TiO_2_. The experimental and theoretical calculation results suggest that the introduced O defects play a dual role in accelerating the photocatalytic N_2_ reduction efficiency. An optimized concentration of O defects as electron acceptors can increase the charge separation efficiency. On the other hand, N_2_ adsorbed on OVs can be dissociated and activated through the transfer of electrons into the antibonding orbital of N_2_, thus weakening the strong triple bond [34]. Thus, it is desirable to optimize N_2_ reduction performance by introducing O defect active sites on hollow-structure photocatalysts.

WO_3_ has been widely adopted as a promising photocatalyst due to its appropriate band gap (~2.4 eV), low conduction band edge potential, and fleet electron transport rate [35,36,37]. Moreover, the adjustable crystal structure makes the O defect-rich WO_3_ the hotspot in N_2_ reduction. For instance, Wang and coworkers have reported that the light-induced O defects at the grain boundaries of porous WO_3_ can greatly enhance photocatalytic N_2_ reduction performance [38]. Mechanistic studies reveal that the O defect regulates the band structure of WO_3_, providing sufficient driving force to trigger N_2_ reduction. Moreover, O defects can serve as active sites to chemisorb N_2_ molecules. Under light, the chemisorbed N_2_ molecules accepted photoexcited electrons to generate N_2_H* intermediates with coupled protons, further hydrogenating into NH_3_. Chen and coworkers proposed the modification of Fe single atoms to adjust the electronic structure of O-defective WO_3_ and facilitate the adsorption and conversion of N_2_ [39]. These works have revealed that the introduction of the O defect is an efficient approach for promoting photocatalytic N_2_ reduction in thermodynamics, but the synergistic effects of the O defect and hollow structure to further promote N_2_ reduction in dynamics have not been reported.

Herein, we design a series of WO_3_ hollow spheres with O defects (WO_3−x_-HS) to accomplish efficient photocatalytic N_2_ reduction into NH_3_ in H_2_O. The as-synthesized WO_3−x_-HS demonstrates a superior NH_3_ evolution rate of 140.1 μmol g^−1^ h^−1^, which is 7.96 times higher than that of pristine bulk WO_3_. The experimental analysis reveals that the uniform hollow sphere structure provides WO_3−x_-HS with a relatively larger specific surface area and a confined cavity for the adsorption and enrichment of N_2_ molecules on the photocatalyst, while the O defect promotes the activation of the inert N≡N triple bond.

## 2. Results and Discussion

**Figure 1:**
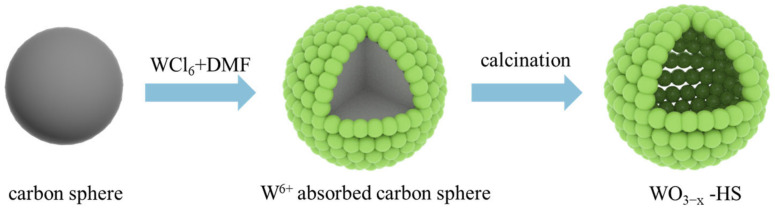
Schematic illustration of the synthesis of WO_3−x_-HS.

The schematic synthetization of WO_3−x_-HS is illustrated in Figure 1. Specifically, the carbon sphere has been first prepared with glucose as a precursor via a hydrothermal process. Then, W^6+^ is adsorbed onto the surface of the carbon sphere. The mixture is calcined at 450 °C to remove the template carbon sphere, and the hollow sphere structure WO_3−x_-HS is obtained. The crystal structures of WO_3−x_-HS and bulk WO_3_ are investigated via X-ray diffraction (XRD). The diffraction peaks of both samples can be matched well with monoclinic tungsten trioxide (PDF#20-1324) (Figure S1). The characteristic peaks at 23.1°, 23.7°, 24.1°, 28.8°, 33.3°, 33.6°, and 34.0° correspond to the (001), (020), (200), (111), (021), (201), and (220) planes of monoclinic WO_3_, respectively.

**Figure 2:**
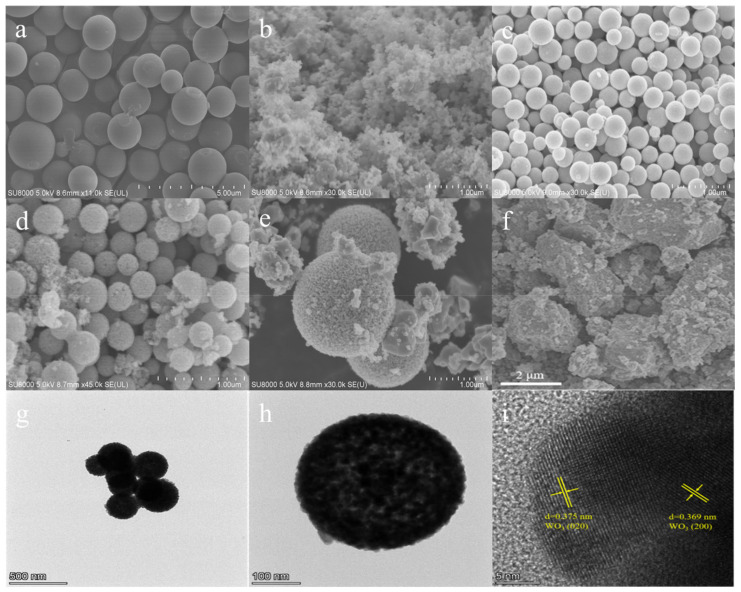
SEM images of carbon sphere (**a**). WO_3−x_-HS samples synthesized with various amounts of carbon sphere template: 0.1 g (**b**), 0.25 g (**c**), 0.4 g (**d**), 0.5 g (**e**), and WO_3_-bulk (**f**). TEM (**g**,**h**) and HRTEM images (**i**) of WO_3−x_-HS.

The morphologies of the as-synthesized samples are carefully investigated via scanning electron microscopy (SEM) and transmission electron microscopy (TEM). As displayed in Figure 2a, the obtained carbon sphere exhibits a typical and uniform spherical morphology. Figure 2b–e presents the WO_3−x_-HS samples synthesized with different amounts of template. When a 0.1 g carbon sphere is used, the obtained sample shows an amorphous structure, which may be due to the insufficient template. With the increasing amount of carbon sphere, the hollow sphere structure formed gradually. WO_3−x_-HS synthesized with the addition of a 0.25 g template presents the most uniform and complete hollow sphere morphology. The surface is clear and smooth. As a counterpart, the WO_3_-bulk exhibits an irregularly shaped block structure consisting of small particles (Figure 2f). The TEM image (Figure 2g) further reveals the sphere structure of WO_3−x_-HS. Moreover, the brightness of the outer edge of the WO_3−x_-HS is lower than center position, indicating that the prepared WO_3−x_-HS is a hollow sphere with a cavity structure (Figure 2h). The high-resolution TEM (HRTEM) of WO_3−x_-HS is shown in Figure 2i. Distinct 0.375 nm and 0.369 nm lattice fringes are observed, which correspond to the (020) and (200) crystal planes of tungsten oxide, respectively. Moreover, the elemental mapping results reveal the co-existence of W and O with uniform dispersion in WO_3−x_-HS (Figure S2).

**Figure 3:**
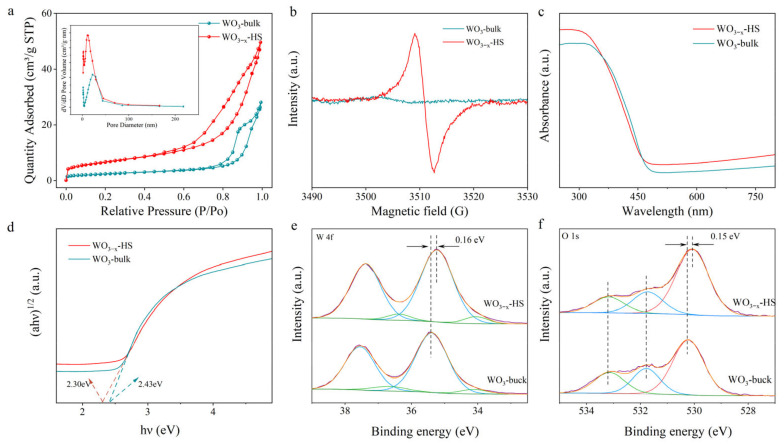
BET (**a**), EPR (**b**), DRS (**c**), Tauc plots (**d**), high-resolution XPS spectra of W 4f (**e**) and O 1s (**f**) of WO_3−x_-HS and WO_3_-bulk.

It is well known that the photocatalytic performance is closely related to the specific surface area size and porosity of the catalyst [40,41]. The N_2_ adsorption/desorption isotherms and the pore size distribution of the obtained WO_3−x_-HS and WO_3_-bulk products are measured and presented in Figure 3a. Both catalysts have strong interactions with N_2_ in the low-pressure region and show H3-type hysteresis loops in the high-pressure region, indicating that both samples have type IV adsorption–desorption isotherms [42]. The Brunauer–Emmett–Teller (BET) surface areas of WO_3−x_-HS and WO_3_-bulk are determined to be 23.71 m^2^ g^−1^ and 8.53 m^2^ g^−1^, respectively. The larger surface areas of WO_3−x_-HS are expected to expose more catalytically active sites and enhance the contact and adsorption of nitrogen molecules. The pore size distribution curves confirm the presence of mesopores (2–50 nm) in both samples, and the porosities of WO_3−x_-HS and WO_3_-bulk are 0.077 cm^3^ g^−1^ and 0.043 cm^3^ g^−1^, respectively. The larger porosity of WO_3−x_-HS can accelerate the transport of reactants and products during N_2_ photocatalytic nitrogen fixation, providing more opportunities for proton capture and the deep hydrogenation of intermediates in photocatalytic nitrogen fixation. The presence of oxygen vacancy is verified using electron paramagnetic resonance (EPR). As can be seen in Figure 3b, no signal is observed for WO_3_-bulk in the whole range. While a significant resonance peak at g-factor value of 2.003 ascribed to the unpaired electrons is observed, suggesting the existence of abundant O vacancies in WO_3−x_-HS [23]. The possible O vacancy formation mechanism may be that the removal of the reductive carbon template induces the escape of lattice oxygen. The optical properties of the obtained photocatalysts have been evaluated using diffuse reflectance spectroscopy (DRS). As shown in Figure 3c, the absorption edge of WO_3−x_-HS shows a blue-shift when compared with WO_3_-bulk. Moreover, WO_3−x_-HS exhibits stronger light absorption properties in the visible light region, which may be due to the existence of a “sub-band” induced by O vacancies. The band energies (E_g_ value) of the catalysts are calculated using Tauc fitting curves (Figure 3d) [43]. The E_g_ values of WO_3−x_-HS and WO_3_-bulk are determined to be 2.30 and 2.43 eV, respectively.

The surface chemical state of elements of as-synthesized samples has been evaluated using an X-ray photoelectron spectroscope (XPS) (Figure 3e,f). The W 4f spectra of both samples are deconvoluted into two doublet peaks, associated with two different states of the W element. The peaks in WO_3_-bulk at the binding energies at 35.39 and 37.55 eV correspond to the W 4f_7/2_ and W 4f_5/2_ signals of W^6+^. The second doublet peaks at 34.12 and 36.67 eV are assigned to the W 4f_7/2_ and W 4f_5/2_ of W^5+^. Note that the W 4f binding energies of WO_3−x_-HS are lower than those of WO_3_-bulk, indicating a decrease in electron density after the generation of O vacancies [44]. The O 1s spectrum of WO_3_-bulk can be fitted with three peaks at the binding energies of 530.22, 531.78, and 533.15 eV, which are attributed to the lattice oxygen, O in the surface hydroxyl group, and chemisorbed oxygen, respectively [45]. There is a decrease in the binding energy of lattice oxygen in WO_3−x_-HS when compared to that of WO_3_-bulk, which is attributable to the increased electron cloud density caused by the formation of O vacancies.

**Figure 4:**
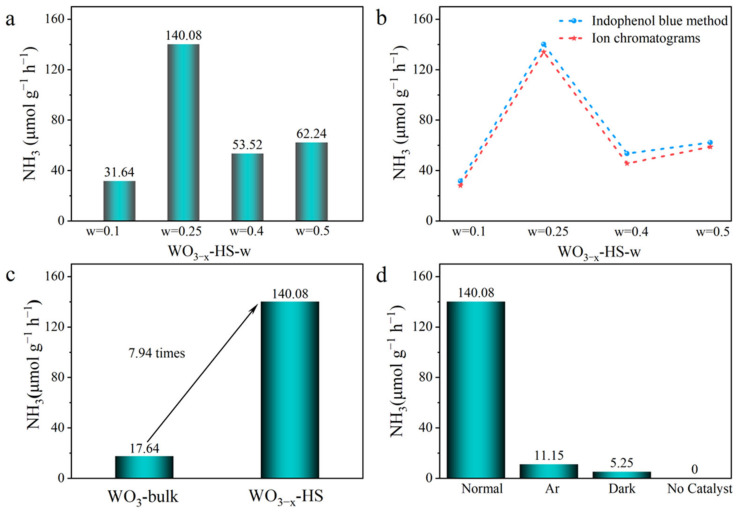
Photocatalytic N_2_ reduction performance of WO_3−x_-HS-w prepared with different amounts of carbon sphere as template (**a**); comparison of two methods for determining ammonia concentration (**b**); comparison of photocatalytic N_2_ reduction performance of WO_3−x_-HS and WO_3_-bulk (**c**); control experiments of WO_3−x_-HS for photocatalytic N_2_ reduction performance under different conditions (**d**).

The photocatalytic nitrogen reduction reaction (NRR) activities of the as-synthesized samples have been evaluated with H_2_O and N_2_ as feedstocks. Figure 4a shows the performance of WO_3−x_-HS prepared with different amounts of carbon sphere as a template. It is interesting to see that the NH_3_ generation rates exhibit a trend of increasing initially and decreasing afterwards with the increasing amount of carbon sphere. The WO_3−x_-HS synthesized with the addition of a 0.25 g template presents the optimal NRR activity, with an ammonia production rate of 140.08 μmol g^−1^ h^−1^. It can also be seen that the prepared WO_3−x_-HS exhibits better performance than most of the reported works on photocatalytic N_2_ reduction (Table S1). It seems to match with the SEM results that a higher NRR performance is achieved with a more complete hollow sphere structure. The high performance of NRR over WO_3−x_ is also confirmed by ion chromatography (Figure 4b). To further reveal the role of hollow structure and O vacancies in effecting the photocatalytic performance, the NRR activity of WO_3_-bulk is tested. As displayed in Figure 4c, the WO_3_-bulk exhibits a relatively low NH_3_ generation rate of 17.64 μmol g^−1^ h^−1^. The possible reasons may be that the hollow sphere structure of WO_3−x_-HS with a larger specific surface area exposes more active sites of O vacancies to activate the N≡N. The hollow structure with a nanoconfined cavity promotes the reaction kinetics. The photocatalytic performance of WO_3−x_-HS under different reaction conditions has been investigated. As can be seen from Figure 4d, when the gas is changed from N_2_ to Ar, almost no ammonia is produced. At the same time, ammonia could not be detected without a catalyst or in dark conditions. All these results elucidate that the evolved NH_3_ is indeed originated from N_2_ through the photocatalytic reduction process.

The WO_3−x_-HS synthesized with the addition of 0.25 g of template showed the best NRR activity with an ammonia generation rate of 140.08 μmol g^−1^ h^−1^. The prepared WO_3−x_-HS outperforms most of the reported research results in photocatalytic reduction of N_2_ (Table S1). This seems to be in agreement with the SEM results that a more complete hollow sphere structure can achieve higher NRR performance.

**Figure 5:**
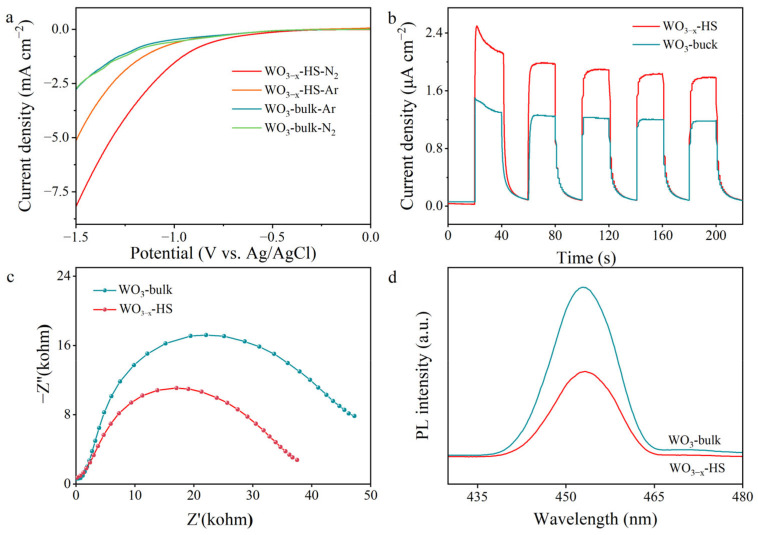
LSV curves (**a**), transient photocurrent responses (**b**), EIS Nyquist plots (**c**), and PL spectra (**d**) of WO_3−x_-HS and WO_3_-bulk.

Linear scanning voltammetry (LSV) tests have been performed on WO_3−x_-HS and WO_3_-bulk in Ar- and N_2_-saturated electrolyte environments to evaluate the NRR performance. A distinct current density enhancement under N_2_ over Ar suggests a potential NRR process driven by both samples (Figure 5a). The photocurrent densities of WO_3−x_-HS in a N_2_ environment are much higher than those of WO_3_-bulk, indicating better NRR activity [46]. Electrochemical characterizations have been performed to reveal the charge transfer and separation behaviors. Figure 5b shows the transient photocurrent response of the sample. The photocurrent density of WO_3−x_-HS is significantly higher compared to WO_3_-bulk, indicating that the WO_3−x_-HS material is more capable of photo-induced electron–hole pair separation [47]. Electrochemical impedance spectroscopy (EIS) is used to monitor charge transfer behavior. As shown in Figure 5c, WO_3−x_-HS presents a decreased semicircle compared to the WO_3_-bulk, indicating lower electron transfer resistance [48]. These results demonstrate that the O vacancies on WO_3−x_-HS contribute to the improved separation and transfer of photoinduced carriers. The evaluation of carrier separation efficiency is conducted via steady-state PL emission spectroscopy. As shown in Figure 5d, under excitation light at a wavelength of 453 nm, there is an obvious PL quenching of WO_3−x_-HS compared to WO_3_-bulk, indicating the photogenerated electron–hole recombination is suppressed and the photogenerated carrier utilization efficiency is higher [49]. The reason for this result may be that the hollow structure with a relatively thin shell of WO_3−x_-HS is beneficial for the migration rate and separation efficiency of photogenerated carriers.

**Figure 6:**
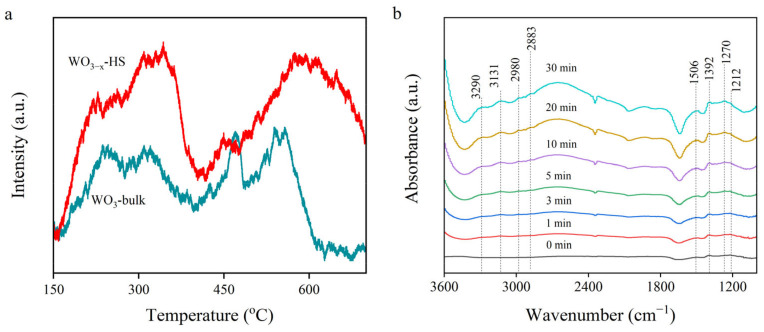
N_2_-TPD of WO_3−x_-HS and WO_3_-bulk (**a**); in situ DRIFTS spectra of WO_3−x_-HS during photocatalytic N_2_ fixation process (**b**).

The N_2_ adsorption performance of the catalysts was characterized using N_2_ temperature-programmed desorption spectrometry (N_2_-TPD). As shown in Figure 6a, both WO_3−x_-HS and WO_3_-bulk show adsorption peaks at low (230–360 °C) and high temperatures (470–650 °C), which are attributed to the physical adsorption and chemical adsorption peaks of N_2_, respectively [50]. The peak intensities of WO_3−x_-HS are stronger than those of WO_3_-bulk, and the peak position shows a positive shift to the relatively high temperature, which certifies the much-improved N_2_ affinity. These results reveal that the designed hollow sphere structure with confined inner micro-space and O vacancies are beneficial for the adsorption of N_2_ molecules and for facilitating the NRR process.

In situ diffuse reflectance infrared Fourier transform spectroscopy (in situ DRIFTS) has been tested to reveal the possible N_2_ reduction process. The WO_3−x_-HS catalyst is exposed to a vapor of N_2_ and water to simulate the reaction conditions. The in situ DRIFTS is recorded with different light irradiation times. As shown in Figure 6b, the peak at 1506 cm^−1^ can be attributed to the OH bending mode of adsorbed water [51]. When light is introduced, the newly emerged peak at 3290 cm^−1^ is attributed to the ν(N-H) stretching mode in the reacting intermediate [52]. The signals at about 1212, 1270, and 1392 cm^−1^ are assigned to bending vibrations of ammonia species and intermediates σ(N-H), while the peaks at about 2883 and 2980 cm^−1^ are absorption peaks of NH_4_^+^ [51,53]. Moreover, these peaks intensified with prolonged irradiation time, revealing that N_2_ is gradually hydrogenated to ammonia over the catalyst consistently.

**Figure 7:**
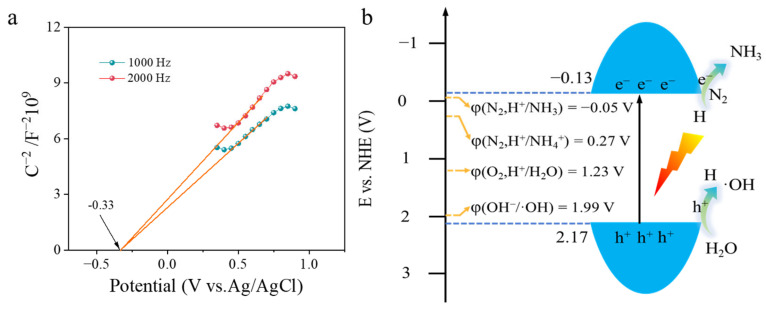
Mott–Schottky plots of WO_3−x_-HS (**a**) and a schematic illustration of the band structure of WO_3−x_-HS (**b**).

Mott–Schottky tests were performed to evaluate the energy band positions of WO_3−x_-HS (Figure 7a). The slope of the linear potential curves of WO_3−x_-HS is positive, indicating that the as-synthesized sample is an n-type semiconductor material [54]. The flat band potentials of WO_3−x_-HS are determined to be −0.33 V (vs. Ag/AgCl, pH = 7), corresponding to −0.133 V vs. NHE based on the equation (E_NHE_ = E_Ag/AgCl_ + 0.197) [55]. The conduction band of n-type semiconductors is 0–0.1 V higher than the flat-band potential. Here, the voltage difference between the conduction band (CB) and the flat potential is set to 0 V; thus, the minimum conduction band (CB) for WO_3−x_-HS is about −0.13 V (vs. NHE, pH = 7). Combined with the DRS result, the valence bands (VB) of WO_3−x_-HS are calculated to be 2.17 V (vs. NHE, pH = 7) [56]. Thus, the band structure of WO_3−x_-HS is shown in Figure 7b. Apparently, it is thermodynamically favorable for the CB electrons to reduce N_2_ into NH_3_ and VB holes to oxidize H_2_O.

A possible NRR mechanism for WO_3−x_-HS has been proposed. In the reaction system, N_2_ molecules are confined and enriched in the inner cavity of WO_3−x_-HS. Beneficiating from the thin shell of the hollow structure, O vacancies in WO_3−x_-HS are sufficiently exposed as active sites. Thus, the activation of the N≡N triple bond is achieved by the electron-rich environment of O vacancies via the π-back-donation into N_2_ antibonding orbitals. Upon irradiation, photogenerated holes on the VB of WO_3−x_-HS oxidize H_2_O into O_2_ and release active protons. The electrons accumulated on the CB reduce the activate *N_2_ into NH_3_.

## 3. Materials and Methods

### 3.1. Materials and Reagents

Tungsten chloride (WCl_6_) was purchased from Aladdin. Glucose (C_6_H_12_O_6_·H_2_O), cetyltrimethylammonium bromide (C_19_H_42_BrN), sodium tungstate dihydrate (Na_2_WO_4_·2H_2_O), N, N-dimethylformamide (C_3_H_7_NO), and ethanol (C_2_H_6_O) were from Sinopharm Chemical Reagent Co., Ltd. (Shanghai, China). Nitric acid (HNO_3_) was purchased from Xilong Science Co. (Shantou, Shantou, China). Deionized water (18.2 MΩ cm) was obtained from a waterproof system and used in all experiments. All chemicals used in this work were of analytical grade. None of the above reagents required further purification.

### 3.2. The Preparation of Carbon Nanosphere

Carbon nanospheres were prepared by glucose under hydrothermal conditions. An amount of 6 g of glucose was dissolved in 6 mL of deionized water. The solution was transferred to a 100 mL of PTFE reactor and reacted at 180 °C for 12 h in an oven. After the hydrothermal reaction was completed, the supernatant was centrifugally washed with deionized water and ethanol until the ionic solubility of the supernatant was less than 10 ppm. The final product was dried in a vacuum oven at 40 °C for 12 h.

### 3.3. The Preparation of WO_3−x_-HS

WO_3−x_-HS was prepared by controlling the gradual hydrolysis of WCl_6_ on the template carbon sphere particles. WCl_6_ was used as the metal precursor and dissolved into N, N-dimethylformamide (DMF) with a concentration of 0.1 g mL^−1^ denoted as solution A. At the same time, various amounts of carbon nanospheres (0.1, 0.25, 0.4, and 0.5 g) and 0.1 g of cetyltrimethylammonium bromide (CTAB) were dispersed into 50 mL of DMF solution and denoted as solution B. Afterwards, solution A was dropped into solution B under continuous ultrasonic treatment for 30 min. After that, 1 mL of deionized water was added dropwise to make the WCl_6_ hydrolyze. The suspension was stirred at room temperature for 24 h, then washed with anhydrous ethanol and water, and finally freeze-dried. The obtained product was calcined in a muffle furnace at 450 °C for 2 h and marked as WO_3−x_-HS.

### 3.4. The Preparation of Bulk WO_3_

Firstly, 4 g of Na_2_WO_4_·2H_2_O was dissolved in 20 mL water to obtain a homogeneous solution, then 50 mL HNO_3_ solution (1 mol L^−1^) was dropped into the above solution and stirred for another hour. The precipitate was washed with deionized water and ethanol and dried in a vacuum oven at 60 °C. Finally, the dried sample was grounded and calcined at 450 °C for 2 h, denoted as WO_3_-bulk.

### 3.5. Photocatalytic N_2_ Fixation

The photocatalytic nitrogen fixation reaction was carried out at room temperature by first adding the photocatalyst (0.015 g) and 50 mL of deionized water to a quartz reactor with a quartz top and sonicating for 3 min. The reactor was then evacuated and subsequently bubbled with ultrapure N_2_ for 30 min in the darkness. Finally, the reaction was carried out under the illumination of a 300 W xenon lamp (CEL-HXF300-T3 with filter (420 nm < λ < 800 nm)) for 1 h with continuously bubbling N_2_ in the whole reaction process. A quantity of the reaction solution was collected, filtered, and then assayed for ammonia concentration by the indophenol blue method. The average ammonia concentration was calculated by three parallel experiments. The calibration curve of NH^4+^ measured by UV-vis spectra is displayed in Figure S3.

### 3.6. Characterization

The crystal structure of the photocatalysts was analyzed by X-ray electron diffraction on a Bruker D8 Advance X-ray diffractometer. The morphological and lattice structure information was characterized using scanning electron microscopy (Hitachi SU8000, Tokyo, Japan) and transmission electron microscopy (FEI Talos F200s, Hillsboro, OR, USA). Ultraviolet-visible diffuse reflectance spectroscopy was measured on a Varian Cary 500 UV-vis spectrophotometer. The chemical states of the prepared samples were characterized via X-ray photoelectron spectroscopy on a VG Scientific ESCA Lab Mark II spectrometer. The binding energies of all tested elements were calibrated by C 1s at 284.6 eV. Electron paramagnetic resonance measurements were performed on a Bruker A300 EPR spectrometer. Electrochemical tests were performed on a ZENNIUM IM6 electrochemical workstation (Zahner, Kronach, Bayern, Germany). The chemisorption properties of the catalyst for N_2_ were characterized using N_2_ temperature-programmed desorption spectrometry on an AutoChem II 2920 (Micromeritics) instrument with a thermal conductivity detector. PL was measured by a fluorophotometer (Edinburgh FLS1000) with an excitation wave length of 453 nm.

### 3.7. Electrochemistry Measurement

Fluorine-doped tin oxide (FTO) glass was used for the preparation of the working electrode. The FTO glass was first washed by sonication in acetone and ethanol for 30 min. Next, 5 mg of the as-synthesized samples were added to 0.5 mL N, N-dimethylformamide. The mixture was continuously sonicated for 2 h to obtain a uniform slurry. Then, 10 μL of the obtained homogeneous slurry was dropped onto the FTO side with exposed areas of 0.25 cm^2^. The remaining parts that were not coated were sealed with epoxy resin. Finally, the electrode was dried in nature. A conventional three-electrode cell was used for the electrochemical tests. A Pt plat and a Ag/AgCl electrode were adopted as counter electrodes and reference electrodes, respectively. Na_2_SO_4_ aqueous was used as an electrolyte.

## 4. Conclusions

In summary, we have developed a WO_3−x_ hollow sphere with oxygen defects for efficient N_2_ photoreduction into NH_3_. It is revealed that the hollow sphere structure greatly promotes the enrichment of N_2_ molecules in the inner cavity and facilitates the chemisorption of N_2_ onto WO_3−x_-HS. O vacancies exposed on the surface accelerate the activation of the N≡N triple bond. Moreover, the thin shell of the hollow structure facilitates the photogenerated charge transfer. As such, the WO_3−x_-HS exhibits better photocatalytic NRR activity with an optimal NH_3_ generation rate of 140.08 μmol g^−1^ h^−1^, which is 7.94 times higher than the counterpart WO_3_-bulk. This work validates an avenue for designing active photocatalysts toward N_2_ reduction via morphology and structure regulation.

## Data Availability

Data are contained within the article and Supplementary Materials.

## Supplementary Materials

The following supporting information can be downloaded at: https://www.mdpi.com/article/10.3390/molecules28248013/s1, Figure S1: XRD pattern of WO_3−x_-HS and WO_3_-bulk; Figure S2: HAADF-STEM (a) and corresponding elemental mapping images W (b) and O (c) of WO_3−x_-HS; Figure S3: The calibration curve of NH_4_^+^ reference by UV-vis spectra at room temperature; Table S1: Comparison of photocatalytic N_2_ reduction performance among reported catalysts. References [57,58,59,60,61,62,63,64,65,66,67] are citation in the Supplementary Materials.
