# Supplementary material for: Synergistic Spatial Confining Effect and O Vacancy in WO3 Hollow Sphere for Enhanced N2 Reduction
Source: Molecules. 2023 Dec 8;28(24):8013. doi: 10.3390/molecules28248013 (PMC10745342; doi:10.3390/molecules28248013)
Supplement: Supplementary file 1 [file molecules-28-08013-s001.zip › molecules-2742863-supplementary.pdf]

Supporting Information

Synergistic Spatial Confining Effect and O Vacancy in WO_3 Hollow Sphere for Enhanced N_2 Reduction

Yuzhou Xia [1,2,3,†], Xinghe Xia [1,2,†], Shuying Zhu [1,*], Ruowen Liang [2], Guiyang Yan [2], Feng Chen [2,*] and Xuxu Wang [3]

1 College of Chemistry, Fuzhou University, Fuzhou 350116, China; yzxia@ndnu.edu.cn (Y.X.); 211327039@fzu.edu.cn (X.X.)

2 Fujian Province University Key Laboratory of Green Energy and Environment Catalysis, Ningde Normal University, Ningde 352100, China; rwliang@ndnu.edu.cn (R.L.); ygyfjnu@163.com (G.Y.)

3 State Key Laboratory of Photocatalysis on Energy and Environment, Research Institute of Photocatalysis, College of Chemistry, Fuzhou University, Fuzhou 350116, China; xwang@fzu.edu.cn

* Correspondence: syzhu@fzu.edu.cn (S.Z.); t9309@ndnu.edu.cn (F.C.)

† These authors contributed equally to this work.

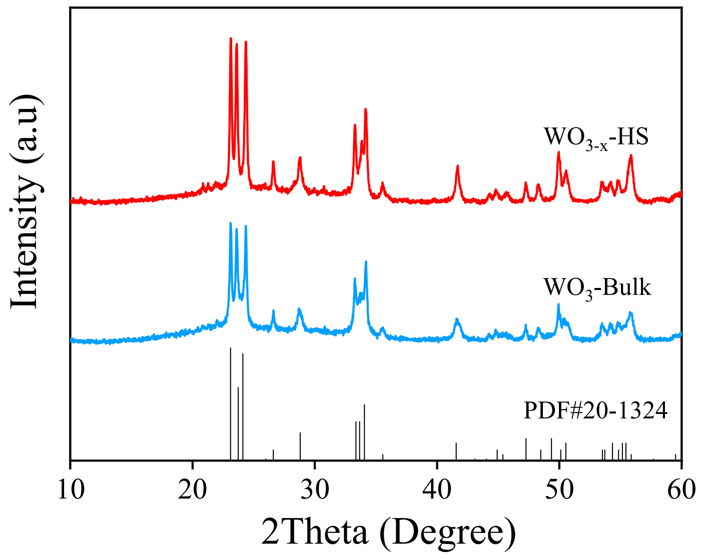

Figure S1. XRD pattern of WO_{3-x}-HS and WO_3-bulk.

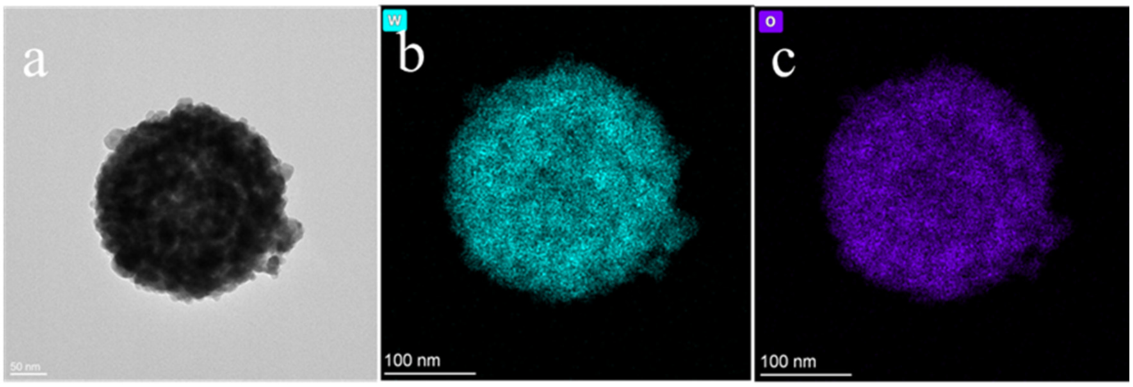

Figure S2. HAADF-STEM (a) and corresponding elemental mapping images W (b) and O (c) of WO_{3-x}-HS.

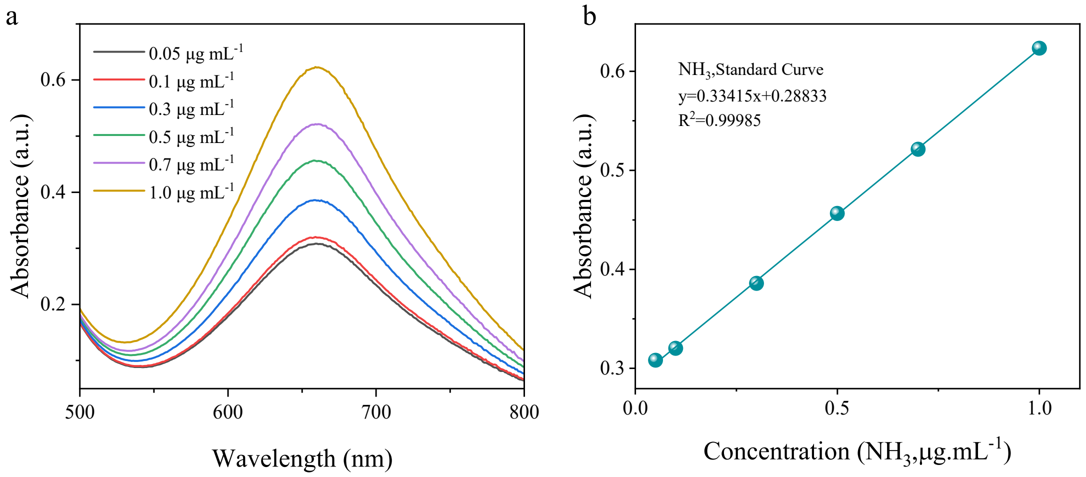

Figure S3. The calibration curve of NH_4^+ reference by UV-vis spectra at room temperature.

Table S1. Comparison of photocatalytic N_2 reduction performance among reported catalysts.

Catalyst	Light source	Reaction medium	NH_3 yield rate (μmol g^{-1} h^{-1})	Ref.
WO_{3-x} -HS	300 W Xe lamp, $\lambda \geq 420$ nm	Water	140.1	This work
WO_{3-x}	300 W Xe lamp	Methanol (12.5 vol%) aqueous solution	28.4	1
NP WO_{3-x}	LED lamp (4 × 3 W)	Na_2SO_3(1 mM) aqueous solution	82.41	2
OVs-BWO	300 W Xe lamp, λ<420 nm	Water	106.4	3
MoO_{3-x}/Fe-$W_{18}O_{49}$	300 W Xe lamp	Water	137.5	4
NH_2-MIL-125 (Ti)	300 W Xe lamp, $\lambda > 400$ nm	Water	12.3	5
In_2O_3/In_2S_3	300 W Xe lamp	Water	40.04	6

FPx	300 W Xe lamp	Water	62.42	7
Cu_2O/MIL-100(Fe)	300 W Xe lamp, $\lambda > 400$	Water	51.22	8
SMO-10	300 W Xe lamp	Water	3.92	9
Ru-TiO_2 NS	300 W Xe lamp	Ethanol (20 vol%) aqueous solution	3.31	10
NTO-0.5	300 W Xe lamp, $\lambda > 400$	Water	80.09	11
P-Fe/$W_{18}O_{49}$	Xenon lamp	Na_2SO_3(1 mM) aqueous solution	187.6	12
1 mol% Mo/$W_{18}O_{49}$	300 W Xe lamp, $\lambda \geq 400$ nm	Na_2SO_3(1 mM) aqueous solution	195.5	13
BCN	250 W Xe lamp, $\lambda \geq 400$ nm	Na_2SO_3(1 mM) aqueous solution	313.9	14
BiCNx-5	300 W Xe lamp, $\lambda > 420$ nm	Na_2SO_3(1 mM) aqueous solution	576.11	15

Reference

1. Shi, K.; Wang, F.; Li, X.; Huang, W.; Lu, K.; Yu, C.; Yang, K., Defect-engineered WO_{3-x} nanosheets for optimized photocatalytic nitrogen fixation and hydrogen production. *Journal of Materials Science* **2023,** 58, (42), 16309-16321.

2. Yang, Z.; Wang, J.; Wang, J.; Li, M.; Cheng, Q.; Wang, Z.; Wang, X.; Li, J.; Li, Y.; Zhang, G., 2D WO_{3-x} Nanosheet with Rich Oxygen Vacancies for Efficient Visible-Light-Driven Photocatalytic Nitrogen Fixation. *Langmuir* **2022,** 38, (3), 1178-1187.

3. Wang, T.; Feng, C.; Liu, J.; Wang, D.; Hu, H.; Hu, J.; Chen, Z.; Xue, G., Bi_2WO_6 hollow microspheres with high specific surface area and oxygen vacancies for efficient photocatalysis N_2 fixation. *Chemical Engineering Journal* **2021,** 414.

4. Wang, L.; Wang, S.; Cui, D.; Li, M.; Yang, X.; Li, F.; Xu, L., Z-scheme heterojunctions with double vacancies semiconductors MoO_{3-x} and Fe-doped $W_{18}O_{49}$ for photocatalytic nitrogen fixation. *Journal of Alloys and Compounds* **2022,** 927.

5. Huang, H.; Wang, X.-S.; Philo, D.; Ichihara, F.; Song, H.; Li, Y.; Li, D.; Qiu, T.; Wang, S.; Ye, J., Toward visible-light-assisted photocatalytic nitrogen fixation: A titanium metal organic framework with functionalized ligands. *Applied Catalysis B: Environmental* **2020,** 267.

6. Xu, H.; Wang, Y.; Dong, X.; Zheng, N.; Ma, H.; Zhang, X., Fabrication of

In_2O_3/In_2S_3 microsphere heterostructures for efficient and stable photocatalytic nitrogen fixation. *Applied Catalysis B: Environmental* **2019,** 257.

7. Hu, T.; Jiang, G.; Yan, Y.; Lan, S.; Xie, J.; Zhang, Q.; Li, Y., Facile synthesis of Fe single-atom porous photocatalysts via direct metal atomization achieving efficient photocatalytic nitrogen fixation. *Journal of Materials Science & Technology* **2023,** 167, 248-257.

8. Huang, X.; Shi, Y.; Liu, C.; Wang, Z.; Bi, J.; Yu, J. C.; Wu, L., Enhanced photocatalytic nitrogen fixation on Cu_2O clusters/MIL-100(Fe) heterojunction. *Applied Surface Science* **2023,** 640.

9. Li, Q.; Zhao, Z.; Bai, X.; Tong, X.; Yue, S.; Luo, J.; Yu, X.; Wang, Z.; Wang, Z.; Li, P.; Liang, Y.; Wang, Z., Tunable and stable localized surface plasmon resonance in $SrMoO_4$ for enhanced visible light driven nitrogen reduction. *Chinese Journal of Catalysis* **2021,** 42, (10), 1763-1771.

10. Liu, S.; Wang, Y.; Wang, S.; You, M.; Hong, S.; Wu, T.-S.; Soo, Y.-L.; Zhao, Z.; Jiang, G.; Jieshan, Q.; Wang, B.; Sun, Z., Photocatalytic Fixation of Nitrogen to Ammonia by Single Ru Atom Decorated TiO_2 Nanosheets. *ACS Sustainable Chemistry & Engineering* **2019,** 7, (7), 6813-6820.

11. Li, C.; Gu, M.; Gao, M.; Liu, K.; Zhao, X.; Cao, N.; Feng, J.; Ren, Y.; Wei, T.; Zhang, M., N-doping TiO_2 hollow microspheres with abundant oxygen vacancies for highly photocatalytic nitrogen fixation. *Journal of Colloid and Interface Science* **2022,** 609, 341-352.

12. Dong, G.; Huang, X.; Bi, Y., Anchoring Black Phosphorus Quantum Dots on Fe-Doped $W_{18}O_{49}$ Nanowires for Efficient Photocatalytic Nitrogen Fixation. *Angewandte Chemie International Edition* **2022,** 61, (30).

13. Zhang, N.; Jalil, A.; Wu, D.; Chen, S.; Liu, Y.; Gao, C.; Ye, W.; Qi, Z.; Ju, H.; Wang, C.; Wu, X.; Song, L.; Zhu, J.; Xiong, Y., Refining Defect States in $W_{18}O_{49}$ by Mo Doping: A Strategy for Tuning N_2 Activation towards Solar-Driven Nitrogen Fixation. *Journal of the American Chemical Society* **2018,** 140, (30), 9434-9443.

14. Wang, W.; Zhou, H.; Liu, Y.; Zhang, S.; Zhang, Y.; Wang, G.; Zhang, H.; Zhao, H., Formation of B-N-C Coordination to Stabilize the Exposed Active Nitrogen Atoms in g-C_3N_4 for Dramatically Enhanced Photocatalytic Ammonia Synthesis Performance. *Small* **2020,** 16, (13).

15. Liang, C.; Niu, H.-Y.; Guo, H.; Niu, C.-G.; Yang, Y.-Y.; Liu, H.-Y.; Tang, W.-W.; Feng, H.-P., Efficient photocatalytic nitrogen fixation to ammonia over bismuth monoxide quantum dots-modified defective ultrathin graphitic carbon nitride. *Chemical Engineering Journal* **2021,** 406.
